# Gausemycin A-Resistant *Staphylococcus aureus* Demonstrates Affected Cell Membrane and Cell Wall Homeostasis

**DOI:** 10.3390/microorganisms11051330

**Published:** 2023-05-18

**Authors:** Darya V. Poshvina, Diana S. Dilbaryan, Alexey S. Vasilchenko

**Affiliations:** Laboratory of Antimicrobial Resistance, Institute of Environmental and Agricultural Biology (X-BIO), Tyumen State University, 625003 Tyumen, Russia; dvposhvina@mail.ru (D.V.P.); d.d.s98@mail.ru (D.S.D.)

**Keywords:** gausemycin A, lipoglycopeptide, peptide antibiotics, antimicrobial peptide, *Staphylococcus aureus*, antibiotic resistance

## Abstract

Antibiotic resistance is a significant and pressing issue in the medical field, as numerous strains of infectious bacteria have become resistant to commonly prescribed antibiotics. *Staphylococcus aureus* is a bacterium that poses a grave threat, as it is responsible for a large number of nosocomial infections and has high mortality rates worldwide. Gausemycin A is a new lipoglycopeptide antibiotic that has considerable efficacy against multidrug-resistant *S. aureus* strains. Although the cellular targets of gausemycin A have been previously identified, detailing the molecular processes of action is still needed. We performed gene expression analysis to identify molecular mechanisms that may be involved in bacterial resistance to gausemycin A. In the present study, we observed that gausemycin A-resistant *S. aureus* in the late-exponential phase showed an increased expression of genes involved in cell wall turnover *(sceD*), membrane charge (*dltA*), phospholipid metabolism (*pgsA*), the two-component stress-response system *(vraS*), and the Clp proteolytic system (*clpX*). The increased expression of these genes implies that changes in the cell wall and cell membrane are essential for the bacterial resistance to gausemycin A. In the stationary phase, we observed a decrease in the expression of genes involved in the phospholipid metabolism (*mprF*) and Clp proteolytic system (*clpX*).

## 1. Introduction

The issue of antibiotic resistance among pathogenic microorganisms is reaching alarming levels worldwide. This growing problem poses a significant threat to public health as it reduces the effectiveness of antibiotics in treating infectious diseases [1]. *Staphylococcus aureus* is a major human pathogen that causes serious infections worldwide, often with severe consequences, including high morbidity and mortality [2]. Of particular concern is *S. aureus’s* high capacity to acquire and accumulate mechanisms of antibiotic resistance. As a result, the emergence of drug-resistant strains of *S. aureus* is becoming an increasingly significant public health concern. The rise in antibiotic resistance among Gram-positive pathogens, including *S. aureus*, has driven the development of new antibiotics that are effective against multidrug-resistant (MDR) strains. One promising approach is the use of peptide antibiotics [3], which offer a unique mode of action and are less likely to induce resistance in bacteria. Consequently, peptide antibiotics have the potential to provide a valuable therapeutic option for combating MDR pathogens like *S. aureus*. Gausemycin A is a novel member of the lipoglycopeptide family of natural peptide antibiotics produced by *Streptomyces* sp. INA-As-5812 [4]. It exhibits significant activity against Gram-positive bacteria, including methicillin-resistant *S. aureus* (MRSA), which is notorious for its high level of antibiotic resistance. The proposed mechanism includes disruption of the integrity of the bacterial membrane in a Ca-dependent manner, making it similar to daptomycin [5,6]. The precise mechanism of resistance to gausemycin A is not yet fully understood, although certain features of the resistance phenotype have been identified. Previous research has demonstrated that *S. aureus* is capable of developing resistance to gausemycin A [7], which is characterized by an additional reduction in susceptibility to daptomycin [7]. Further studies are necessary to better understand the resistance profile of gausemycin A and its implications for the treatment of multidrug-resistant Gram-positive infections.

The molecular mechanisms that control the integrated response of a microbial cell to antibiotic-induced stress may be associated with changes in the expression of one gene or a group of genes that ensure cell survival and proliferation. For this, candidate genes were selected that could potentially be involved in the formation of resistance to gausemycin A. The *dltABCD* operon plays a significant role in the addition of D-alanine to teichoic acids in Gram-positive bacteria [8]. Mutations in the *dlt* operon or alterations in the expression of its genes can lead to an increase in the positive charge on the cell surface of *S. aureus*. This phenomenon is due to modifications in the composition of teichoic acids, which are involved in the regulation of cell wall integrity and resistance to antimicrobial peptides. These changes may confer resistance to antibiotics such as daptomycin. 

The *sceD* gene encodes a lytic transglycosylase (SceD) that plays a crucial role in the control of cell wall expansion, remodeling, and daughter cell separation. Additionally, SceD is involved in peptidoglycan turnover, which is essential for the maintenance of cell wall integrity and the regulation of bacterial shape. Consequently, alterations in SceD function can affect these critical processes, potentially leading to changes in cell morphology and antibiotic susceptibility. In particular, autolysin cleaves the cell wall in such a way as to support the integrity of the cell wall during cell division [9]. A proteomic study showed that the amount of SceD protein was increased in the cell wall fraction of strains with reduced sensitivity to vancomycin, and changes in its expression and activity were responsible for changes in the rate of cell wall turnover and changes in the peptidoglycan structure [10]. 

The *walK* gene, also known as *yycG*, encodes for the synthesis of a histidine kinase sensor and belongs to the two-component regulatory system WalR/WalK (YycF/YycG). This system has an impact on the synthesis of several Gram-positive proteins, including proteins involved in cell wall metabolism and permeability [11]. The *mprF* gene encodes lysylphosphatidylglycerol synthetase (MprF), a bifunctional bacterial enzyme that synthesizes the positively charged lipid lysyl-phosphatidylglycerol (LysPG) and translocates it subsequently from the inner membrane leaflet to the outer membrane leaflet by the flippase domain of the MprF protein, causing an increased net positive charge on the cytoplasmic membrane [12,13]. Eventually, this process leads to an increase in the positive surface charge of the cell membrane and can serve as a protective barrier against antibiotic binding [14]. When cell wall biosynthesis is inhibited by antibiotics, *S. aureus* responds by rapidly activating a group of genes called “cell wall stress stimulon”. Some of these genes are controlled by the three-component system VraTSR. The *vraS* and *vraR* genes play a critical role in regulating antibiotic resistance.

In addition, resistance to gausemycin A may be associated with genes encoding enzymes involved in phospholipid metabolism, such as phosphatidylglycerol and cardiolipin synthetases (PgsA and Cls, respectively) [15]. The Clp proteolytic system is a molecular mechanism in *S. aureus* that plays an important role in both virulence and environmental adaptation of bacteria. The ClpX is an ATP-dependent specificity component of the ClpXP protease, which regulates numerous intracellular proteins in the stability of cell wall hydrolases, thereby controlling cell wall metabolism [16,17].

The purpose of this study was to explore the quantitative expression levels of several genes involved in different aspects of bacterial cell physiology, including cell wall turnover (*sceD*), membrane charge regulation (*mprF* and *dltA*), phospholipid metabolism (*pgsA*), two-component regulatory systems responsible for maintaining cell envelope homeostasis (*walK, vraS*) and the proteolytic system (*clpX*). This investigation was conducted to better understand the molecular mechanisms underlying the resistance of gram-positive bacteria to gausemycin A and other lipoglycopeptides.

## 2. Materials and Methods

### 2.1. Bacterial Strains, Media, and Growth Conditions

*S. aureus* GAU-S (Gausemycin A-susceptible) and *S. aureus* GAU-R (gausemycin A-resistant) strains were grown in Mueller Hinton Broth with 50 mg/mL CaCl_2_ in the absence of gausemycin A. Cells were collected at mid-exponential, late-exponential, and stationary growth phases, then diluted to an optical density corresponding to 10^6^ CFU/mL. 

### 2.2. Minimum Inhibitory Concentration

The minimum inhibitory concentration (MIC) was determined following the Clinical and Laboratory Standards Institute guidelines in the 96-well plate, containing 90 μL of inoculum prepared in growth media at 10^6^ CFU/mL with 10 μL of two-fold dilutions of the antibiotics. The plates were incubated for 24 h without shaking at 37 °C. All experiments were performed in biological triplicates. The bacterial growth was assessed by scanning the absorbance data at 600 nm obtained using a spectrophotometer (Multiscan GO, Thermo Fisher Scientific, Waltham, MA, USA). 

### 2.3. Spa Typing and MLST

The procedure was carried out as previously described [18]. The Spa types have been defined using spaTyper 1.0 (https://cge.cbs.dtu.dk/services/spatyper, accessed on 30 April 2023). The MLST STs were assigned the publicly available MLST server (https://cge.cbs.dtu.dk/services/MLST, accessed on 30 April 2023). The characteristics of the strains are shown in Table 1.

### 2.4. RNA Extraction and Reverse Transcription into cDNA

Total RNA was extracted using the RNeasy Mini kit (Qiagen) according to the manufacturer’s instructions. Total RNA concentration was assessed using Qubit 4.0 (Thermo Fisher Scientific), and the quality of the RNA extracted was estimated using TapeStation 4150 (Agilent Technologies, Santa Clara, CA, USA). The Agilent TapeStation 4150 (Agilent Technologies, USA) system, which is an automated instrument for nucleic acid gel electrophoresis, assigns RNA Integrity Number (RIN) values ranging from 1 to 10, with 10 being the highest quality. Only samples with preserved 16S and 23S peaks and RIN values > 8 were selected for gene expression analyses. The RIN values > 8 indicate intact, high-quality RNA samples for downstream applications [19]. Total RNA was further treated with DNase I (New England Biolabs, Ipswich, MA, USA) followed by the RNA Clean & lConcentrator-5 kit (Zymo Research, Seattle, WA, USA), according to the instruction manual. DNase I-treated RNA from each sample was reverse-transcribed to cDNA using the iScript reversed transcription supermix for RT-qPCR reagent (Bio-Rad, Hercules, CA, USA) in accordance with the manufacturer’s protocol. 

### 2.5. Gene Expression Analysis

qRT-PCR was performed with a Roche LightCycler 96 system using an SsoAdvanced Universal SYBR Green Supermix (Bio-Rad Laboratories). RT-PCR reaction mix with a volume of 20 µL was prepared with 300 nM of each primer (final concentration) and 50 ng of template RNA. A protocol with the following thermal cycling conditions was used: DNA denaturation at 95 °C for 3 min, followed by 40 cycles of denaturation at 95 °C for 10 s and annealing/extension at 60 °C for 15 s. After the last amplification cycle, a melting-curve analysis was carried out with heating from 65 to 95 °C in increments of 0.5 °C/s. Negative controls (without template or reverse transcriptase enzyme) were included in each run. The characteristics of the primers of both reference and target genes are reported in Table S1. Gene-specific were designed using the online service “Integrated DNA technologies” (https://www.idtdna.com/Primerquest/Home/Index, accessed on 30 April 2023) and gene sequences present in the GenBank database. Fold changes in the gene expression levels were normalized in relation to the levels of *gyrB* mRNA. The relative changes in gene expression were quantified using the Pfaffl method [20]:

gene expression ratio = (E_target_)^∆Cttarget(control-sample)^/(E_reference_)^∆Ct reference (control-sample)^,

where E_target_ is the amplification efficiency of target (gene of interest), E_reference_ is the amplification efficiency of reference (*gyrB*), Ct is the point at which the fluorescence rises above the background fluorescence, ∆Ct_target_ is the Ct deviation of the control minus the sample of the target gene transcript, and ∆Ct_reference_ is the Ct deviation of the control minus the sample of the reference gene transcript. 

### 2.6. Statistical Analysis

The results obtained were analyzed using the Origin 2021 software (OriginLab Corporation, Northampton, MA, USA) and statistically evaluated. The significance of the differences between the pairs was determined using the paired-sample Student’s *t*-test.

## 3. Results 

### 3.1. Comparison of Gene Expression between the GAU-S and GAU-R Strains

The relative quantitative expression as a ratio of transcripts of GAU-R versus its parent strain GAU-S was evaluated during mid-exponential, late-exponential, and stationary growth phases in drug-free conditions using RT-qPCR. During the mid-exponential phase, a marked increase in gene expression was observed in GAU-R compared to GAU-S. Specifically, the genes *vraS* (a two-component sensor histidine kinase), *dltA* (a subunit of the D-alanine–poly(phosphoribitol) ligase), and *clpX* (an ATP-dependent Clp protease ATP-binding subunit) showed a 4.53, 8.71, and 6.23-fold increase in expression, respectively (see Table 2). 

In addition, several other genes showed significant upregulation in GAU-R relative to GAU-S during the mid-exponential phase. These included *mprF* (a bifunctional lysyl-phosphatidylglycerol flippase/synthetase), *walK* (a cell wall metabolism sensor histidine kinase), and *pgsA* (a CDP-diacylglycerol-glycerol-3-phosphate 3-phosphatidyltransferase). Conversely, the gene encoding transglycosylase (*sceD*) was found to be downregulated in GAU-R compared to GAU-S during this same phase. Notably, the genes *vraS, pgsA*, and *clpX* continued to show significant upregulation in GAU-R during the late-exponential phase, with fold changes of 6.19, 8.31, and 10.25, respectively (Table 2). 

Conversely, during the stationary growth phase, both *mprF* and *clpX* showed a statistically significant reduced expression in GAU-R compared to GAU-S, with fold changes of 5.52 and 6.77, respectively (see Table 2). Overall, the results indicate that the gausemycin A-resistant variant exhibits an increased expression of *vraS*, *pgsA*, *dltA*, and *clpX* genes relative to its parent gausemycin A-susceptible strain.

### 3.2. The Gene Expression Depending on Growth Phase

**Figure 1:**
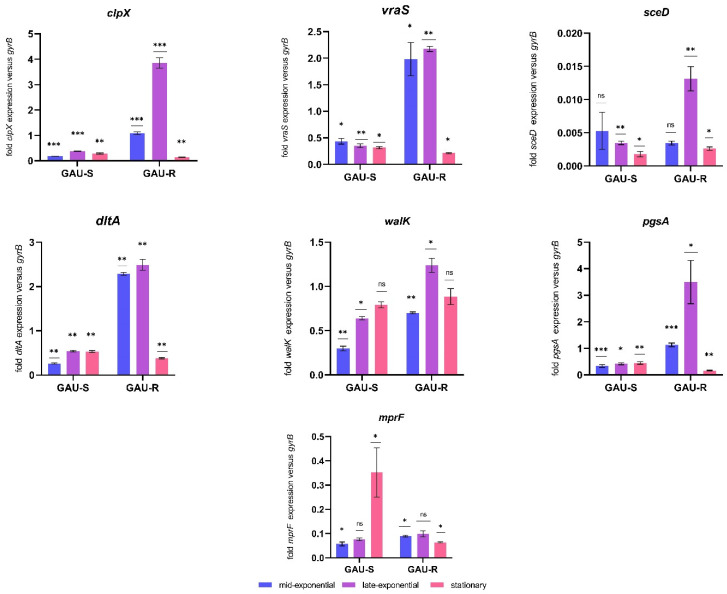
Expression analysis of genes: *mprF* (phosphatidylglycerol lysyltransferase), *pgsA* (CDP-diacylglycerol-glycerol-3-phosphate 3-phosphatidyltransferase), *sceD* (transglycosylase), *dltA* (D-Alanine–poly(phosphoribitol) ligase subunit 1), *vraS* (sensor histidine kinase), *clpX* (ATP-dependent Clp protease ATP-binding subunit), *walK* (cell wall metabolism sensor histidine kinase). Transcript levels of the target genes were determined with RT-qPCR in relation to *gyrB* (DNA gyrase subunit B) expression. All values presented in this study represent the mean of three independent replicate cultures for each strain, and error bars indicate the standard deviation of comparisons between the replicates. The growth phases analyzed were mid-exponential, late-exponential, and stationary. (* *p* < 0.05, ** *p* < 0.005, *** *p* < 0.0005, ns—not significant). *p*-Value was derived by comparing the gene expression of GAU-R relative to its parent strain GAU-S.

Bacterial growth is subject to dynamic changes in cell density, nutrient availability, pH, and other physical and chemical factors. To account for these changes, we analyzed growth-phase-dependent gene expression profiles (Figure 1). In the GAU-susceptible strain, we observed that *vraS* exhibited the highest transcript level during the mid-exponential phase, with decreasing transcriptional activity as growth progressed. Conversely, the highest and lowest transcriptional activity of *clpX* was observed during the late-exponential and stationary growth phases, respectively. Interestingly, we observed that the transcript level of *walK* increased from the mid-exponential phase to the stationary growth phase. Additionally, *mprF* and *pgsA* showed the highest transcriptional activity during the stationary growth phase, while *sceD* was most active during the mid-exponential phase. Furthermore, we observed that *dltA* expression was elevated from the mid-exponential phase to the stationary growth phase (Figure 1). However, it should be noted that the differences in expression levels between the growth phases were not always statistically significant. 

In the GAU-resistant strain, all of the studied genes exhibited the highest transcript level during the late-exponential phase, with significantly reduced transcriptional activity in the stationary phase. Notably, the differences in expression levels between growth phases were not always statistically significant for *vraS* and *mprF* (Supplementary Table S2). These results suggest that the growth phase strongly influences gene expression.

## 4. Discussion

The molecular mechanisms underlying the development of a gausemycin A-resistant phenotype and cross-resistance to daptomycin are not well understood. In our previous study, after 20 passages, the mutant strain *S. aureus* GAU-R was characterized by an increase in the MIC of gausemycin A from 2.5 to 200 µg/mL [7]. In the present study, we aimed to elucidate the mechanism underlying the acquisition of gausemycin A resistance by *S. aureus*. Our results demonstrate that this process is complex and involves changes in the expression of genes involved in cell wall and cell membrane homeostasis. 

Two-component systems (TCSs) are a primary strategy used by bacteria to adapt to changing environments via signal transduction. It was previously established that genetic changes in two-component regulatory systems are strongly associated with phenotypes resistant to peptide antibiotics [21]. WalRK (also called YycFG) is a regulatory system controlling the expression of genes involved in cell wall metabolism, thereby controlling autolysis, biofilm formation, and virulence [22,23]. Previous studies have shown that overexpression of the WalKR two-component regulatory system positively regulates differential gene expression related to cell wall integrity (*lytM* and *sceD*), surface charge (*mprF* and *dltABCD*), poly-glucosamine biofilm formation (*icaAD*), and downregulation of cell-wall-associated proteins (CWAPs) such as Coa and ClfB [24,25,26,27]. According to the results of Howden B.P. et al. (2011), the impacts of the single substitutions in either WalR or WalK dramatically change the bacterial cell physiology, with significant reductions in autolytic activity and increases in cell wall thickness linked to the insertion of WalR or WalK alleles [28]. The *walK* gene is known to positively regulate differential gene expression related to cell wall integrity, surface charge, biofilm formation, and downregulation of certain proteins. Our study found a 2.35-fold increase in *walK* gene expression during the mid-exponential growth phase and a 1.93-fold increase during the late-exponential growth phase. These results are consistent with a study by Kuroda et al. (2019), which reported increased expression of *walK* in vancomycin-resistant *S. aureus* [21].

The expression of the *sceD* gene, which encodes lytic transglycosylase, was upregulated in GAU-R compared with GAU-S, with a 3.78-fold increase in the late-exponential growth phase. This finding is consistent with the results of McEvoy et al. (2013), who also observed increased expression of *sceD* for vancomycin-resistant *S. aureus* [29]. Therefore, we suggest that the expression of genes involved in cell wall metabolism could play a critical role in the development of the GAU-R phenotype.

Another tactic used by *S. aureus* to increase the positive surface charge is to increase the expression of the *dlt* and *mprF* genes [30]. The *dltABCD* operon contributes to the net positive surface charge by d-alanylating wall teichoic acids through distinct effector mechanisms [31]. We observed the increased expression of the *dltA* gene for GAU-R in the mid-exponential growth phase (8.71-fold, Table 2). The multiple-peptide resistance factor (MprF) is a membrane protein that consists of a C-terminal enzymatic domain responsible for aminoacylating the headgroup of phosphatidylglycerol (PG) and a hydrophobic N-terminal domain responsible for flipping the modified lipid to the external side of the cytoplasmic membrane. The *mprF* gene, similar to the *dlt* operon, contributes to the overall positive cell surface charge involved in the transformation of PG into a positively charged lysyl-PG, followed by its translocation to the outer surface of the cytoplasmic membrane. A correlation between the presence of MprF and a decrease in the activity of peptide antibiotics such as daptomycin (lipopeptide) and vancomycin (glycopeptide) against resistant bacteria was determined. We found markedly increased *mprF* expression in GAU-R in the mid-exponential and in the late-exponential phases (1.56 and 1.30-fold, respectively; Table 2), which is consistent with previous work showing that high expression of *mprF* and *dltABCD* can promote the formation of daptomycin without *mprF* mutation [14,32,33]. Thus, the associated effect of both the *mprF* and *dltABCD* mechanisms could result in reduced access of calcium-gausemycin A complex to its membrane target.

The study also examined another gene involved in the production of membrane phospholipids called CDP-diacylglycerol-glycerol-3-phosphate-3-phosphatidyltransferase (*pgsA*). PgsA, which is an enzyme embedded in the membrane, catalyzes the primary reaction in the biosynthesis of phosphatidylglycerol through the phosphatidylglycerol phosphate (PGP) synthase reaction. Phosphatidylglycerol carries a negative charge, while Lys-PG is positively charged. The enzyme MprF can convert one to the other. The conversion of phosphatidylglycerol to Lys-PG or vice versa can cause an increase in either positive or negative charge, thereby altering the overall charge of the bacterial membrane. This change can enhance bacterial resistance to certain antibiotics, such as cationic antimicrobial peptides, especially daptomycin [34,35]. In the late-exponential phase, the expression level of the *pgsA* gene in the GAU-R strain increased by 8.31-fold, as demonstrated in this study. In our previous work, we also found that the *S. aureus* GAU-R strain had a slightly more negative relative net charge, as measured with zeta potential, compared to the *S. aureus* GAU-S strain [7]. Other studies have reported increased levels of lysyl-PG and decreased levels of PG in daptomycin-resistant isolates [36,37], which sets gausemycin A apart from daptomycin in terms of action.

In addition to changes in the membrane, it is suggested that alterations in the cell wall also contribute to gausemycin A resistance. When cell wall biosynthesis is impeded by either antibiotics or a depletion of the biosynthesis machinery, *S. aureus* responds by quickly activating a group of genes called the cell wall stress stimulon [38]. A subset of these genes is controlled by the three-component system VraTSR [39]. The *Vra*TSR system consists of *Vra*S (sensor histidine kinase), *Vra*R (response regulator), and *Vra*T for the stimulation and activation of *Vra*TSR. *The vraS* and *vraR* genes play a crucial role in the regulation of antibiotic resistance [40]. Activation of the three-component regulatory system VraTSR reduces the sensitivity of staphylococci to vancomycin, daptomycin, and oxacillin [41]. Quantitative analysis of the *vraS* gene expression showed an increase in the level of transcriptional activity by 4.53 times in the mid-exponential phase and 6.19 times in the late-exponential phase for GAU-R compared to GAU-S, as indicated in Table 2.

The Clp proteolytic system is a molecular machinery in *S. aureus* that plays a role in both virulence and environmental adaptation. In recent years, several studies have pointed to a link between Clp proteins and antibiotic resistance. The system consists of the proteolytic subunit ClpP and the ATP-dependent protease ClpX [42]. Specifically, the ClpXP system is responsible for the degradation of multiple proteins within the cell and acts as a global regulator. ClpP and ClpX have been found to affect functions such as low-temperature tolerance, biofilm formation, surface protein production, autoinducer production, autolytic cleavage of daughter cells, high osmolarity growth, toxin production, and hemoglobin binding [43,44,45]. RNA sequencing analysis (RNA-Seq) proves that ClpX has a profound effect on cell physiology and demonstrates that ClpX primarily influences gene expression through ClpXP-dependent pathways. ClpX appears to control the expression of major virulence genes such as *spa* (protein A), *nuc* (nuclease), *geh* (glycerol ester hydrolase), and SAUSA300_1890 (stafopain A protease). Several studies demonstrate that ClpX is important for maintaining resistance to antimicrobial peptides, nisin, and the antibiotics penicillin and daptomycin [46,47,48]. All of these antimicrobials interact with or target the cell wall and/or cell membrane, as does gausemycin A. Our findings are consistent with a recent report by Zou L. et al. (2021), which demonstrated that the loss of ClpXP protease activity leads to a reduction in resistance to antimicrobials targeting the cell envelope [48]. In our study, we observed a more than 10-fold increase in the expression of the *clpX* gene in the GAU-R mutant compared to the wild-type, as measured with qPCR in late-exponential phase cultures (Table 2).

## 5. Conclusions

**Figure 2:**
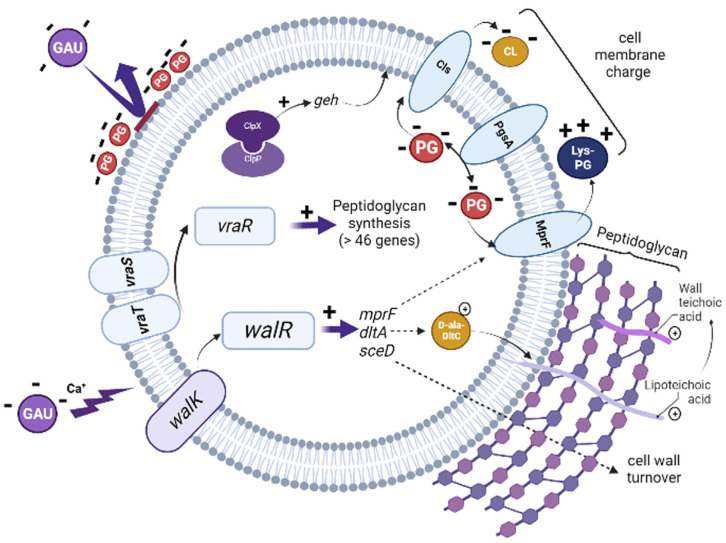
Proposed model for the development of resistance of *S. aureus* FDA209P to gausemycin A. 1. The interaction of the anionic molecule gausemycin A with a negatively charged cell membrane occurs in a Ca^2+^-dependent manner; 2. The action of gausemycin A activates the WalRK and VraTSR regulatory systems: WalR positively regulates the expression of the *mprF*, *dltA*, and *sceD* genes. In turn, VraR activates the expression of genes involved in peptidoglycan biosynthesis. 3. An increase in the expression of the *pgsA* gene, which is involved in the process of phosphatidylglycerol (PG) biosynthesis, increases the overall negative charge of the bacterial cell; 4. A significant increase in the level of transcriptional activity of the *clpX* gene activates the *geh* gene encoding the enzyme Geh (glycerol ester hydrolase), resulting in a change in the profile of membrane fatty acids. The image was created in “BioRender.com”.

Antibiotic resistance among *Staphylococcus aureus* is a worldwide problem. *S. aureus* is a significant threat to human health that has been made worse by its continued development of antibiotic resistance. New antimicrobials and treatment options are needed to combat this, but developing such methods is challenging. Therefore, a better understanding of common antibiotic targets, such as the cell wall and cell membrane, and how these targets change in antibiotic-resistant strains, could provide valuable insights for the development of new antibiotics. As far as we know, this is the first report that provides new insights into the molecular mechanisms that contribute to the resistance of *S. aureus* to the lipoglycopeptide antibiotic gausemycin A. Our data suggest that resistance to gausemycin A is complex and stepwise, and it is currently difficult to isolate specific genes or groups of genes responsible for the development of resistance. However, analysis of the expression profile of selected genes has shown that the mechanisms underlying the development of the gausemycin A-resistant phenotype involve modifications in the structure of the cytoplasmic membrane and cell wall, which are controlled by different two-component regulatory systems (Figure 2).

## Figures and Tables

**Table 1:** Characteristics of the study strains.

*S. aureus* Strains	Spa Type	MLST	MIC µg/mL (Broth Microdilution Method) of Selected Antibiotics	
			Daptomycin	Gausemycin A
GAU-R	t3297	ST464	5.00	>200
GAU-S	t3297	ST464	1.25	2.5

**Table 2:** Fold change in the gene expression of GAU-R relative to its parent strain GAU-S.

Gene	Mid-Exponential Phase	*p*-Value	Late-Exponential Phase	*p*-Value	Stationary Phase	*p*-Value
*vraS*	4.53 up	0.003508	6.19 up	0.000015	1.48 down	0.004713
*mprF*	1.56 up	0.007591	1.30 up	0.060267	5.5 down	0.015934
*sceD*	1.54 down	0.331873	3.79 up	0.002776	1.47 up	0.004713
*dltA*	8.72 up	0.000002	4.58 up	0.000109	0.71 down	0.001534
*walK*	2.35 up	0.000133	1.93 up	0.0011	1.12 up	0.190768
*pgsA*	3.39 up	0.000553	8.32 up	0.007331	0.36 down	0.002019
*clpX*	6.24 up	0.000112	10.25 up	0.000085	6.78 down	0.003211

## Data Availability

Not applicable.

## Supplementary Materials

The following supporting information can be downloaded at: https://www.mdpi.com/article/10.3390/microorganisms11051330/s1, Table S1: Primers used in the RT-qPCR study; Table S2: Fold change in the gene expression of GAU-S and GAU-R during growth phases.

## References

[1] Michael C.A., Dominey-Howes D., Labbate M. (2014). The antimicrobial resistance crisis: Causes, consequences, and management. Front. Public Health.

[2] Lade H., Kim J.-S. (2021). Bacterial Targets of Antibiotics in Methicillin-Resistant *Staphylococcus aureus*. Antibiotics.

[3] Casanova N.G., Ruiz M.S., Bellido J.L.M. (2017). Mechanisms of resistance to daptomycin in *Staphylococcus aureus*. Rev. Esp. Quimioter..

[4] Tyurin A.P., Alferova V.A., Paramonov A.S., Shuvalov M.V., Kudryakova G.K., Rogozhin E.A., Zherebker A.Y., Brylev V.A., Chistov A.A., Baranova A.A. (2021). Gausemycins A, B: Cyclic lipoglycopeptides from *Streptomyces* sp.. Angew. Chem. Int. Ed..

[5] Vasilchenko A.S., Julian W.T., Lapchinskaya O.A., Katrukha G.S., Sadykova V.S., Rogozhin E.A. (2020). A Novel Peptide Antibiotic Produced by *Streptomyces roseoflavus* Strain INA-Ac-5812 with Directed Activity against Gram-Positive Bacteria. Front. Microbiol..

[6] Kravchenko T.V., Paramonov A.S., Kudzhaev A.M., Efimova S.S., Khorev A.S., Kudryakova G.K., Ivanov I.A., Chistov A.A., Baranova A.A., Krasilnikov M.S. (2022). Gausemycin antibiotic family act via Ca^2+^-dependent membrane targeting. ChemRxiv.

[7] Poshvina D.V., Dilbaryan D.S., Kasyanov S.P., Sadykova V.S., Lapchinskaya O.A., Rogozhin E.A., Vasilchenko A.S. (2022). *Staphylococcus aureus* is able to generate resistance to novel lipoglycopeptide antibiotic gausemycin A. Front. Microbiol..

[8] Ernst C.M., Staubitz P., Mishra N.N., Yang S.-J., Hornig G., Kalbacher H., Bayer A.S., Kraus D., Peschel A. (2009). The bacterial defensin resistance protein mprf consists of separable domains for lipid lysinylation and antimicrobial peptide repulsion. PLoS Pathog..

[9] Oshida T., Sugai M., Komatsuzawa H., Hong Y.M., Suginaka H., Tomasz A. (1995). A *Staphylococcus aureus* autolysin that has an Nacetylmuramoyl-L-alanine amidase domain and an endo-beta-N-acetylglucosaminidase domain: Cloning, sequence analysis, and characterization. Proc. Natl. Acad. Sci. USA.

[10] Drummelsmith J., Winstall E., Bergeron M.G., Poirier G.G., Ouellette M. (2007). Comparative proteomics analyses reveal a potential biomarker for the detection of vancomycin-intermediate *Staphylococcus aureus* strains. J. Proteome Res..

[11] Tran T.T., Munita J.M., Arias C.A. (2015). Mechanisms of drug resistance: Daptomycin resistance. Ann. N. Y. Acad. Sci..

[12] Friedman L., Alder J.D., Silverman J.A. (2006). Genetic changes that correlate with reduced susceptibility to daptomycin in *Staphylococcus aureus*. Antimicrob. Agents Chemother..

[13] Yang S.-J., Xiong Y.Q., Dunman P.M., Schrenzel J., François P., Peschel A., Bayer A.S. (2009). Regulation of mprF in daptomycin-nonsusceptible *Staphylococcus aureus* strains. Antimicrob. Agents Chemother..

[14] Sabat A.J., Tinelli M., Grundmann H., Akkerboom V., Monaco M., Del Grosso M., Errico G., Pantosti A., Friedrich A.W. (2018). Daptomycin resistant *Staphylococcus aureus* clinical strain with novel non-synonymous mutations in the *mprF* and *vraS* genes: A new insight into daptomycin resistance. Front. Microbiol..

[15] Fischer A., Yang S.J., Bayer A.S., Vaezzadeh A.R., Herzig S., Stenz L., Girard M., Sakoulas G., Scherl A., Yeaman M.R. (2011). Daptomycin resistance mechanisms in clinically derived *Staphylococcus aureus* strains assessed by a combined transcriptomics and proteomics approach. J. Antimicrob. Chemother..

[16] Bæk K.T., Gründling A., Mogensen R.G., Thøgersen L., Petersen A., Paulander W., Frees D. (2014). β-Lactam resistance in methicillin-resistant *Staphylococcus aureus* USA300 is increased by inactivation of the ClpXP protease. Antimicrob. Agents Chemother..

[17] Kirsch V.C., Fetzer C., Sieber S.A. (2021). Global inventory of ClpP- and ClpX-regulated proteins in *Staphylococcus aureus*. J. Proteome Res..

[18] Aires-de-Sousa M., Boye K., de Lencastre H., Deplano A., Enright M.C., Etienne J., Friedrich A., Harmsen D., Holmes A., Huijsdens X.W. (2006). High interlaboratory reproducibility of DNA sequence based typing of bacteria in a multicenter study. J. Clin. Microbiol..

[19] Fleige S., Pfaffl M.W. (2006). RNA integrity and the effect on the real-time qRT-PCR performance. Mol. Asp. Med..

[20] Pfaffl M.W.A. (2001). New mathematical model for relative quantification in real-time RT—PCR. Nucleic Acids Res..

[21] Kuroda M., Sekizuka T., Matsui H., Ohsuga J., Ohshima T., Hanaki H. (2019). IS256-Mediated Overexpression of the WalKR Two-Component System Regulon Contributes to Reduced Vancomycin Susceptibility in a *Staphylococcus aureus* Clinical Isolate. Front. Microbiol..

[22] Cafiso V., Bertuccio T., Spina D., Purrello S., Campanile F., Di Pietro C., Purrello M., Stefani S. (2012). Modulating activity of vancomycin and daptomycin on the expression of autolysis cell-wall turnover and membrane charge genes in hVISA and VISA strains. PLoS ONE.

[23] Ji Q., Chen P., Qin G., Deng X., Hao Z., Wawrzak Z., Yeo W.S., Quang J.W., Cho H., Luo G.Z. (2016). Structure and mechanism of the essential two-component signal-transduction system WalKR in *Staphylococcus aureus*. Nat. Commun..

[24] Utaida S., Dunman P.M., Macapagal D., Murphy E., Projan S.J., Singh V.K., Jayaswal R.K., Wilkinson B.J. (2003). Genome-wide transcriptional profiling of the response of *Staphylococcus aureus* to cell-wall active antibiotics reveals a cell-wall-stress stimulon. Microbiology.

[25] McCallum N., Spehar G., Bischoff M., Berger-Bachi B. (2006). Strain dependence of the cell wall-damage induced stimulon in *Staphylococcus aureus*. Biochim. Biophys. Acta.

[26] Utaida S., Pfeltz R.F., Jayaswal R.K., Wilkinson B.J. (2006). Autolytic properties of glycopeptide-intermediate *Staphylococcus aureus* Mu50. Antimicrob. Agents Chemother..

[27] Dubrac S., Boneca I.G., Poupel O., Msadek T. (2007). New insights into the WalK/WalR (YycG/YycF) essential signal transduction pathway reveal a major role in controlling cell wall metabolism and biofilm formation in *Staphylococcus aureus*. J. Bacteriol..

[28] Howden B.P., McEvoy C.R.E., Allen D.L., Chua K., Gao W., Harrison P., Bell J., Coombs G., Bennett-Wood V., Porter J.L. (2011). Evolution of Multidrug Resistance during *Staphylococcus aureus* Infection Involves Mutation of the Essential Two Component Regulator WalKR. PLoS Pathog..

[29] McEvoy C.R., Tsuji B., Gao W., Seemann T., Porter J.L., Doig K., Ngo D., Howden B.P., Stinear T.P. (2013). Decreased vancomycin susceptibility in *Staphylococcus aureus* caused by IS256 tempering of WalKR expression. Antimicrob. Agents Chemother..

[30] Bertsche U., Yang S.-J., Kuehner D., Wanner S., Mishra N.N., Roth T., Nega M., Schneider A., Mayer C., Grau T. (2013). Increased cell wall teichoic acid production and D-alanylation are common phenotypes among daptomycin-resistant methicillin-resistant *Staphylococcus aureus* (MRSA) clinical isolates. PLoS ONE.

[31] Bertsche U., Weidenmaier C., Kuehner D., Yang S.-J., Baur S., Wanner S., Francois P., Schrenzel J., Yeaman M.R., Bayer A.S. (2011). Correlation of daptomycin resistance in a clinical *Staphylococcus aureus* strain with increased cell wall teichoic acid production and D-alanylation. Antimicrob. Agents Chemother..

[32] Bayer A.S., Mishra N.N., Cheung A.L., Rubio A., Yang S.J. (2016). Dysregulation of mprF and dltABCD expression among daptomycin-nonsusceptible MRSA clinical isolates. J. Antimicrob. Chemother..

[33] Ma Z., Lasek-Nesselquist E., Lu J., Schneider R., Shah R., Oliva G., Pata J., McDonough K., Pai M.P., Rose W.E. (2018). Characterization of genetic changes associated with daptomycin nonsusceptibility in *Staphylococcus aureus*. PLoS ONE.

[34] Yang B., Yao H., Li D., Liu Z. (2021). The phosphatidylglycerol phosphate synthase PgsA utilizes a trifurcated amphipathic cavity for catalysis at the membrane-cytosol interface. Curr. Res. Struct. Biol..

[35] Zhao W., Róg T., Gurtovenko A.A., Vattulainen I., Karttunen M. (2008). Role of Phosphatidylglycerols in the Stability of Bacterial Membranes. Biochimie.

[36] Jiang J.H., Bhuiyan M.S., Shen H.H., Cameron D.R., Rupasinghe T.W.T., Wu C.M., Le Brun A.P., Kostoulias X., Domene C., Fulcher A.J. (2019). Antibiotic resistance and host immune evasion in *Staphylococcus aureus* mediated by a metabolic adaptation. Proc. Natl. Acad. Sci. USA.

[37] Jones T., Yeaman M.R., Sakoulas G., Yang S.J., Proctor R.A., Sahl H.G., Schrenzel J., Xiong Y.Q., Bayer A.S. (2008). Failures in clinical treatment of *Staphylococcus aureus* infection with daptomycin are associated with alterations in surface charge, membrane phospholipid asymmetry, and drug binding. Antimicrob. Agents Chemother..

[38] Nikolic P., Mudgil P. (2023). The Cell Wall, Cell Membrane and Virulence Factors of *Staphylococcus aureus* and Their Role in Antibiotic Resistance. Microorganisms.

[39] Hu Q., Peng H., Rao X. (2016). Molecular events for promotion of vancomycin resistance in vancomycin intermediate *Staphylococcus aureus*. Front. Microbiol..

[40] Bhowmik D., Das B.J., Hazarika M., Chanda D.D., Bhattacharjee A. (2022). Transcriptional analysis of prsA and vraTS regulatory system in methicillin resistant *Staphylococcus aureus* against oxacillin stress. Indian J. Med. Microbiol..

[41] Gardete S., Kim C., Hartmann B.M., Mwangi M., Roux C.M., Dunman P.M., Chambers H.F., Tomasz A. (2012). Genetic pathway in acquisition and loss of vancomycin resistance in a methicillin resistant *Staphylococcus aureus* (MRSA) strain of clonal type USA300. PLoS Pathog..

[42] Olivares A.O., Baker T.A., Sauer R.T. (2016). Mechanistic insights into bacterial AAA+ proteases and protein-remodelling machines. Nat. Rev. Microbiol..

[43] Claunch K.M., Bush M., Evans C.R., Malmquist J.A., Hale M.C., McGillivray S.M. (2018). Transcriptional profiling of the clpX mutant in *Bacillus anthracis* reveals regulatory connection with the lrgAB operon. Microbiology.

[44] Jensen C., Bæk K.T., Gallay C., Thalsø-Madsen I., Xu L., Jousselin A., Torrubia F.R., Paulander W., Pereira A.R., Veening J.-W. (2019). The ClpX chaperone controls autolytic splitting of *Staphylococcus aureus* daughter cells, but is bypassed by β-lactam antibiotics or inhibitors of WTA biosynthesis. PLoS Pathog..

[45] Frees D., Chastanet A., Qazi S., Sørensen K., Hill P., Msadek T., Ingmer H. (2004). Clp ATPases are required for stress tolerance, intracellular replication and biofilm formation in *Staphylococcus aureus*. Mol. Microbiol..

[46] McGillivra S.M., Ebrahimi C.M., Fisher N., Sabet M., Zhang D.X., Chen Y., Haste N.M., Aroian R.V., Gallo R.L., Guiney D.G. (2009). ClpX contributes to innate defense peptide resistance and virulence phenotypes of *Bacillus anthracis*. J. Innate Immun..

[47] McGillivray S.M., Tran D.N., Ramadoss N.S., Alumasa J.N., Okumura C.Y., Sakoulas G., Vaughn M.M., Zhang D.X., Keiler K.C., Nizet V. (2012). Pharmacological inhibition of the ClpXP protease increases bacterial susceptibility to host cathelicidin antimicrobial peptides and cell envelope-active antibiotics. Antimicrob. Agents Chemother..

[48] Zou L., Evans C.R., Do V.D., Losefsky Q.P., Ngo D.Q., McGillivray S.M. (2021). Loss of the ClpXP Protease Leads to Decreased Resistance to Cell-Envelope Targeting Antimicrobials in *Bacillus anthracis* Sterne. Front. Microbiol..

