# Supplementary material for: Gausemycin A-Resistant Staphylococcus aureus Demonstrates Affected Cell Membrane and Cell Wall Homeostasis
Source: Microorganisms. 2023 May 18;11(5):1330. doi: 10.3390/microorganisms11051330 (PMC10220612; doi:10.3390/microorganisms11051330)
Supplement: Supplementary file 1 [file microorganisms-11-01330-s001.zip › microorganisms-2398200-supplementary.pdf]

Table S1 Primers used in the RT-qPCR study

Gene description (designation)	Primer sequence
DNA gyrase subunit B (*gyrB*)	F: GACGTGGTATCCCAGTTGATATT
	R: ACCATGTAAACCACCAGATACTT
Transglycosylase (*sceD*)	F: GGTACTAATGGAGCAGACATGAG
	R: GTGCTTCAACTTCTTGTGATTGT
Cell wall metabolism sensor histidine kinase (*walK*)	F: GCGCACAATGGTCGTATTTG
	R: TTCATCCCAATCACCGTCTTC
CDP-diacylglycerol–glycerol-3- phosphate 3-phosphatidyltransferase (*pgsA*)	F: GCCAGAGAATTTGCCGTAAC
	R: CCAATGGATCACCTAATAAC
ATP-dependent Clp protease ATP-binding subunit (*clpX*)	F: GTGGTGCCTTTGATGGTATTG
	R: CTGGGCGAATTTGTGCTAATAA
Two-component sensor histidine kinase (*vraS*)	F: AGAACCACCATTAGACCAACAA
	R: AGCAAAGCACGCATTTCTTAAC
D-Alanine–poly(phosphoribitol) ligase subunit 1 (*dltA*)	F: TCCCAAGTGCGACGATTTAC
	R: CTTTCAACGCCAACAGGTAATG
Bifunctional lysyl-phosphatidylglycerol flippase/synthetase (*mprF*)	F: CAATGGTTAGACCACCCGATAA
	R: CGTCAACAATTACACCACAGAAA

Table S2. Fold change in the gene expression of GAU-S and GAU-R during growth phases

Gene	Fold change GAU-S Mid-log vs GAU-S Late-Exp	P value GAU-S Mid-log vs GAU-S Late-Exp	Fold change GAU-S Late-Exp vs GAU-S stationary	P value GAU-S Late-Exp vs GAU-S stationary	Fold change GAU-S Mid-log vs GAU-S stationary	P value GAU-S Mid-log vs GAU-S stationary	Fold change GAU-R Mid-log vs GAU-R Late-Exp	P value GAU-R Mid-log vs GAU-R Late-Exp	Fold change GAU-R Late-Exp vs GAU-R stationary	P value GAU-R Late-Exp vs GAU-R stationary	Fold change GAU-R Mid-log vs GAU-R stationary	P value GAU-R Mid-log vs GAU-R stationary
vraS	1,246 down	0.105722	1,112 down	0.232520	1,387 down	0.034592	1,097 up	0.369732	10,22 down	0.000007	9,319 down	0.002269
mprF	1,328 up	0.045860	4,629 up	0.018009	6,149 up	0.015098	1,108 up	0.277999	1,554 down	0.016026	1,402 down	0.002100
sceD	1,527 down	0.337240	1,952 down	0.007955	2,983 down	0.117119	3,817 up	0.002775	5,036 down	0.002181	1,319 dowp	0.040017
dltA	2,074 up	0.000181	1,018 down	0.553286	2,035 up	0.000228	1,089 up	0.068296	6,554 down	0.000085	6,016 down	0.000003
walK	2,144 up	0.000342	1,237 up	0.006604	2,653 up	0.000273	1,763 up	0.001434	1,398 down	0.014999	1,261 up	0.039324
pgsA	1,258 up	0.105264	1,065 up	0.491694	1,342 up	0.064047	3,09 up	0.015388	21,49 down	0.005809	6,955 down	0.000177
clpX	2,166 up	0.000078	1,339 down	0.011037	1,617 up	0.007103	3,56 up	0.000188	27,326 down	0.000070	7,674 down	**0.000100**
